# Direct DNA Sequencing-Based Analysis of Microbiota Associated with Hematological Malignancies in the Eastern Province of Saudi Arabia

**DOI:** 10.1155/2021/4202019

**Published:** 2021-02-03

**Authors:** Faisal M. Alzahrani, Ali Al-Amri, Saeed Sattar Shaikh, Muzaheed, Amer Ibrahim Alomar, Sadananda Acharya, Maryam Ahmed Aldossary, Fathelrahman Mahdi Hassan

**Affiliations:** ^1^Department of Clinical Laboratory Science, College of Applied Medical Sciences, Imam Abdulrahman Bin Faisal University, Dammam, Saudi Arabia; ^2^Department of Internal Medicine/Oncology, College of Medicine, King Fahd Hospital of the Imam Abdulrahman Bin Faisal University, Dammam, Saudi Arabia; ^3^Department of Public Health, College of Public Health, Imam Abdulrahman Bin Faisal University, Dammam, Saudi Arabia

## Abstract

**Introduction:**

Bloodstream infections (BSI) among patients with hematological malignancies (HM) could predispose them to higher morbidity and mortality for various underlying conditions. Several microorganisms, either pathogenic or opportunistic normal human flora, could cause severe bacteremia and septicemia. While conventional methods have their own limitations, molecular methods such as next-generation sequencing (NGS) can detect these blood infections with more reliability, specificity, and sensitivity, in addition to information on microbial population landscape. *Methodology*. Blood samples from HM patients (*n* = 50) and volunteer blood donor control individuals with no HM (*n* = 50) were subjected to 16S rRNA gene amplification using standard PCR protocols. A metagenomic library was prepared, and NGS was run on a MiSeq (Illumina) sequencer. Sequence reads were analyzed using MiSeq Reporter, and microbial taxa were aligned using the Green Genes library.

**Results:**

82% of the patients showed BSI with Gram-negative bacteria as the most predominant group. *E. coli* comprised a major chunk of the bacterial population (19.51%), followed by *K. pneumoniae* (17.07%). The CoNS and *Viridans Streptococci* groups are 17.07% and 14.63%, respectively. Other major species were *S. aureus* (9.75%), *P. aeruginosa* (7.31%), *A. baumannii* (4.87%), *E. cloacae* (4.87%), and *P. mirabilis* (4.87%). 34.14% of the cases among patients showed a Gram-positive infection, while 14.63% showed polymicrobial infections.

**Conclusion:**

Most of the BSI in patients were characterized by polymicrobial infections, unlike the control samples. Molecular methods like NGS showed robust, fast, and specific identification of infectious agents in BSI in HM, indicating the possibility of its application in routine follow-up of such patients for infections.

## 1. Introduction

Hematological malignancies are cancers of the blood-producing cells. Hematopoietic stem cells undergo a highly regulated and coordinated differentiation and maturation process to produce various blood cells. An aberration in the regulatory pathway confers the cells a neoplastic characteristic clinically presenting as hematological malignancy. Hematological malignancies are various heterogenous conditions resulting from abnormal cell division and proliferation in the bone marrow and the lymphatic system [1]. Hematological malignancies are the 5^th^ most common causes of cancers in Saudi Arabia [2]. Yearly, approximately over a thousand people die from this disease in the country (https://www.who.int). This becomes nearly 10% of deaths due to various cancers. Hematological malignancies include acute and chronic myeloid leukemias, acute and chronic lymphocytic leukemias, and various lymphomas and myelomas. Among cancer patients, particularly with hematological malignancies such as leukemias and lymphomas, bacteremia and bacterial septicemia are a major cause of poor patient outcome [3]. Patients with hematological malignancies have higher risks of contracting bloodstream infections (BSI) owing to immunological dysfunction as a result of chemo and radiotherapy. BSI can be caused by various opportunistic bacteria, viruses, and fungi. Microbial invasion and growth in the bloodstream releases toxins characterized by life-threatening multiorgan failure. Bacterial septicemia is one of the top ten causes of death worldwide and more so in patients with hematological malignancies. Successful management of hematological malignancies includes the proper study of local infections, complications, and antibiotic resistance patterns among the isolated microbial pathogens. However, rapid, reliable, and specific microbial detection methods are limited for the accurate diagnosis of infectious etiology. Conventional culture-based methods take a prolonged time duration and are often challenged with specificity and sensitivity issues. Molecular detection methods such as polymerase chain reaction (PCR), though, are quite popular, and they are associated with problems in interpretation, accuracy, and requirement for high technical inputs. Of late, deoxyribonucleic acid (DNA) sequencing methods have been used widely to deduce the genetic information and identify the causative microorganisms. Next-generation sequencing (NGS) has been very popular for its robustness, speed, accuracy, and large number of samples it can handle at a time. This technology can identify viable, dead, viable but not culturable (VBNC) bacteria, fungi, and viruses in multiple heterogeneous sample pools. Additionally, the NGS sequence can give us information on the microbiome—the complete microbial population structure of the study subject unraveling a plethora of information required for successful treatment, including the antibiotic resistance genes that the organism carries.

Management of suspected BSIs with hematological malignancies with information derived from NGS sequencing can pave the way for improvement in patient care. Microbial infections in patients with hematological malignancies are recurring, and at times, serious problems result in high mortality, morbidity, hospital visits, healthcare costs, and prolonged hospitalization. Prescription of inappropriate antimicrobial drugs during the initial stages of infection might show a high rate of mortality, even after switching to the right antimicrobial drug based on the culture-based antibiogram data. As many as 50-80% of hematological malignant patients contract various microbial infections over the course of disease and chemo/radiotherapy. This represents the exposure of a substantial number of this patient population to the risk of further mortality and morbidity. Hence, it is imperative that treating clinicians have an informed understanding of underlying immune deficiencies associated with particular malignancy, therapy regimen, clinical scenario, diagnostic outcome, and antimicrobial therapy. This could assist the clinicians in predicting the likely microbe encountered in the case and thus formulate an effective diagnostic procedure and initiate the right antimicrobial therapy. Early diagnosis of BSIs and causative organisms facilitates the administration of empiric antibiotics which can drastically reduce secondary infection-related mortality among these patients by almost 50% [4]. Hence, firsthand information on the microbiome of bloodstream infections could help strategize the right antimicrobial therapy for such patients. Currently, for Saudi patients of hematological malignancies, there has been no information available in the literature on the microbial load and population in BSI cases. Hence, this study was taken up to throw light on microbial population structure in BSIs among hematological malignancy patients in the Eastern province of Saudi Arabia. The study is also focused on emphasizing the role of modern NGS sequencing methods in delineating microbial population structure in hematological malignancy. Hence, this study was taken up with the objectives to screen the presence of bacterial infections in the patients of hematological malignancies on the basis of clinical evaluation and to evaluate the causative organism of bacterial infections using Direct DNA sequencing.

## 2. Materials and Methods

### 2.1. Study Subjects

This study was conducted in the eastern province of Saudi Arabia for two years, from March 2017 to February 2019. Patients (*n* = 50) with all types of hematological malignancies were recruited after being clinically diagnosed by an oncologist. Patients with bloodstream infections (BSI) were confirmed by consulting a hemato-oncologist, as a part of routine diagnostic follow-up. Control blood samples—with no signs of hematological malignancies (*n* = 50)—were obtained from blood donors at the blood bank of the same hospital. Participants with a history of antibiotic therapy during the last two weeks were excluded from the study. All the participants filled an informed consent form and were informed about the study and study objectives. Ethical approval was obtained (No. 2016-03-164) from the institutional review board (IRB) of the Imam AbdulRahman bin Faisal University prior to the sample collection.

### 2.2. Sample Processing and DNA Extraction

From the recruited participants, about 5 ml of blood samples were collected by venipuncture by a professional phlebotomist and were immediately transported to the laboratory. The blood samples were deep-frozen at -80°C until further processing. The total bacterial metagenomic DNA from the collected blood was extracted using a commercially available DNA extraction kit—QIAamp *cador* Pathogen Mini Kit (Qiagen, Germantown, USA). The manufacturer's instructions and protocols were followed. Briefly, an aliquot of 400 *μ*l of ethylenediaminetetraacetic acid (EDTA)-anticoagulated peripheral whole blood sample was lysed with 20 *μ*l of proteinase K and 100 *μ*l of digestion buffer in a 2 ml microcentrifuge tube at room temperature. The lysate containing microbial metagenome was allowed to bind to a silica column. The metagenomic DNA was eluted in 30 *μ*l of TE (Tris-EDTA) buffer which was quantified using NanoDrop UV-Vis microspectrophotometer (NanoDrop 2000, ThermoFisher Scientific, Massachusetts, USA). All the procedures were done in a laminar flow chamber using hydrophobic pipette filter tips to avoid external DNA contamination of the samples.

### 2.3. 16S rRNA Metagenomic Library Preparation

The protocols proposed by Fouhy et al. [5] were used with some modifications for the preparation of the metagenomic library. Using 16S rRNA metagenomic sequencing library protocol of Illumina, the V3 and V4 variable regions of 16S ribosomal RNA bacterial gene were amplified as per standard PCR protocols. The primer pairs used for the reaction as described by Klindworth et al. [6]. Primers and overhang adapter sequences were as described by Gyarmati et al. [7] and were procured from Eurofins Genomics, Luxembourg, Belgium. The first stage PCR was run on ProFlex PCR System programmable thermocycler (ThermoFisher Scientific, Massachusetts, USA). The 25 *μ*l reaction mixture contained 2.5 *μ*l of microbial DNA (5 ng/*μ*l), 5.0 *μ*l each of forward primer (1 *μ*M) and reverse primer (1 *μ*M), and 12.5 *μ*l of 2X PCR ready mix (KAPA HiFi HotStart ReadyMix, KAPA Biosystems, Wilmington, USA). Amplification reaction condition was set at initial denaturation of 95°C for 3 mins followed by 24 cycles of 0.5 mins each of denaturation (95°C), annealing of primer sets (55°C), and primer-extension (72°C). The reaction was kept at hold at 4°C after a final extension of 5 minutes at 72°C. The PCR product thus obtained was cleaned up employing AMPureXP bead kit (Beckman-Coulter, California, USA) following manufacturer's instruction manual.

A 2^nd^ stage PCR was run to attach dual indices (NexteraXT Index Kit, Illumina, San Diego, USA) to the amplicons obtained in the previous step. The instructions given in the kit was followed. Briefly, PCR reaction was prepared in a 50 *μ*l reaction mixture containing 5.0 *μ*l each of Nextera XT Index primer 1 and primer 2, 25 *μ*l of PCR ready-mix, and 10 *μ*l of PCR grade water. Amplification conditions were retained the same as earlier, however, with only 8 cycles instead of 25 cycles. The PCR product thus obtained was again purified as mentioned earlier in the first stage reaction.

### 2.4. MiSeq Next-Generation Sequencing Run

Pooled libraries were denatured with 10 *μ*l of 0.2 N NaOH in 1540 *μ*l of hybridization buffer (HT1) and were heat-denatured at 96°C. An internal control PhiX was also maintained along with the sample reactions. MiSeq reagent cartridge was loaded with 5 *μ*l of 4 nM pooled library along with equal volume of 0.2 N NaOH. The cartridge was vortexed briefly, centrifuged at 280 × g at room temperature for 1 min followed by incubation at room temperature for the dsDNA to denature to ssDNA. Finally, prechilled 990 *μ*l of HT1 butter was added to 10 *μ*l of denatured DNA and was kept ready for loading the cartridge to the MiSeq sequencing platform using 2 × 300 cycle V3 using the standard protocol.

### 2.5. Data Analysis

Secondary analysis on the system, MiSeq Reporter software was used for deducing microbiome data using comprehensive workflow mode. The metagenomic workflow was selected, which classified all the organisms based on V3 and V4 regions using the database of 16s rRNA data in the system—http://greengenes.lbl.gov/. Sequence reads < 30 bp and quality scores < 30 were excluded in the Fastx toolkit. Paired-end reads are merged and filtered against the human genome build Hg_19. Statistical analysis was performed using Mann–Whitney *U* test with a significance of 0.05.

## 3. Results

The present study recruited 50 hematological malignancy patients with a total of 41 bloodstream infection cases comprising of 9 (21.95%) acute myeloid leukemia (5 males and 4 females), 10 (24.39%) chronic myeloid leukemia (4 males and 6 females), 15 (36.58%) acute lymphoid leukemia (9 males and 6 females), 5 (12.19%) chronic lymphoid leukemia (4 males and 1 female), and 1 (2.43%) each male patient of non-Hodgkin lymphoma and Hodgkin lymphoma. The age group ranged from 18 to 74 years with a median value of 41 years. Total leukocytes raged from 10 to 3800 cells/*μ*l of blood, while neutrophils ranged from 5 to 850 cells/*μ*l (Table 1). As many as 50 blood samples from healthy blood donors were considered as control samples (nonhematological malignant samples), out of which 4 individuals showed the presence of natural flora-associated Gram-positive bacteria in the sequence analysis. None of them had clinical symptoms of bloodstream infections.

## 4. Sequence Reads

MiSeq-based next-generation sequences for one hundred samples resulted in 954,546 reads assigned to bacterial operational taxonomic units (OTUs). As much as 82% of the reads were unmapped, while 16.50% of the reads were mapped to the human genome. The remaining 1.50% of the reads were mapped for different taxonomic groups of microorganisms. All 41 bloodstream infection HM patients and 4 of control samples were positive for various microbial taxa. No microbial detection occurred in non-BSI HM patients and 46 of control samples (blood donor samples).

## 5. Microbial Landscape in Hematological Malignancy

In the entire data set, the phyla Proteobacteria that includes mainly the genus of *Pseudomonas* and *Escherichia* showed the maximum population among patients of hematological malignancy and other phyla represented fewer OTUs. Firmicutes comprising of mostly Gram-positive normal flora such as Staphylococcus were more common in control samples. Groups of Actinobacteria, Fusobacteria, and Bacteroides were insignificant in both samples.

**Figure 1:**
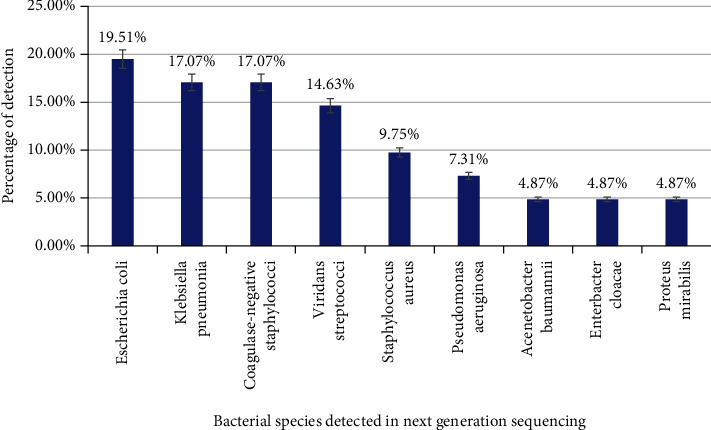
Distribution of bacterial reads among hematological malignancy patients.

From HM patients with BSI, as many as 9 predominantly detected organisms were mapped to the database (Figure 1). An opportunistic *Enterobacteriaceae* family member, *Escherichia coli*, comprised a major chunk of the bacterial population (19.51%), followed by *Klebsiella pneumoniae* (17.07%). Coagulase-negative *Staphylococcus* and Viridans *Streptococci* accounted for 17.07% and 14.63%, respectively. Other major species identified were *Staphylococcus aureus* (9.75%), *Pseudomonas aeruginosa* (7.31%), *Acinetobacter baumannii* (4.87%), *Enterobacter cloacae* (4.87%), and *Proteus mirabilis* (4.87%) (Figure 1).

**Figure 2:**
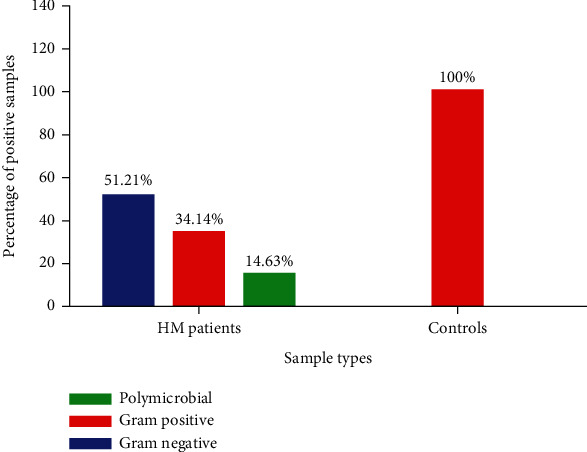
Polymicrobial bacterial infections in patients with hematological malignancies.

Of the total number of reads, among the bloodstream infections in patients, 51.21% of the cases had Gram-negative infection, while control samples had no such findings. About 34.14% of the cases among patients showed Gram-positive infection, while 14.63% of cases showed polymicrobial bloodstream infections. In the case of control samples, only Gram-positive infection was found in all cases (100%) (Figure 2). Most (90%) of the identified reads represented opportunistic human pathogens in hematological malignancy patients, while normal human microbiota in control samples (Table 2).

## 6. Types of Hematological Malignancy and Microbial Groups

The distribution of bacterial bloodstream infections among various types of hematological malignancies in the samples is analyzed. Chronic lymphoid leukemia showed the highest percentage of both Gram-negative infections (42.86%) and Gram-positive infections (35.71%) than all other types of leukemias. These patients were also detected with polymicrobial infection in 50% of cases. Chronic myeloid leukemia had the second-highest rate of bloodstream infection with 33.33% Gram-negative and 28.57% of Gram-positive infection with 33.33% case rate of polymicrobial infections. Control samples (blood donors with no hematological malignancies) showed only Gram-positive infections with no signs of Gram-negative or polymicrobial infections (Figure 2).

## 7. Discussion

The present study recorded as many as 41 out of 50 samples (82%) to be positive for a bloodstream infection among patients of hematological malignancies. This prevalence rate is similar to other studies where hematological cancer patients showed 89% BSI among the patients [8]. Nørgaard [9] opined that BSI are very common in various leukemias, indicating over 90% of patients with infection. Further, all the 41 cases of clinically diagnosed patients showed positivity for the high rate of infection in next-generation sequencing, corroborating the specificity of the modern techniques. However, though control samples had no clinically detected BSIs showing, four of the samples revealed the presence of various Gram-positive normal flora-associated microorganisms. This positivity could be attributed to the contamination of normal skin flora while venipuncture, as the organisms associated are skin-borne natural flora. Chang et al. [10] also showed that skin-associated microbial contamination of blood samples during venipuncture is common and could give false-positive reactions in blood culture and molecular methods. In a study by Jacob et al. [11], the most common hematological malignancy was acute myeloid leukemia, while our study showed that the most common ones are acute lymphatic leukemia (36.58%). However, our small sample size could be the reason for this variation in the type of leukemias recorded.

Proteobacterial members *Escherichia coli* (19.51%) and *Klebsiella pneumoniae* (17.07%) were detected at a higher rate among patients of hematological malignancy. These Enterobacteriaceae family of members are best known for bloodstream infection-causing agents among individuals of compromised immunity. Several researchers have earlier showed that *E. coli* and *Klebsiella pneumoniae* are commonly isolated in patients with hematological malignancy cases [12, 13]. These opportunistic pathogens could get into the bloodstream during various medical interventions of chemo/radiotherapy procedures, as they are part of normal gut microbes.

Members of the Micrococcaceae family coagulate negative Staphylococci, and *Viridans Streptococci* being normal flora of the skin and oral cavity were highly detected at the rate of 17.07% and 14.63%, respectively. Blijlevens et al. [14] and coworkers earlier showed that it is highly common to encounter skin-borne and oral cavity organisms to be in bloodstream infections as a result of iatrogenic transmission of the organism. Several other researchers had earlier detected coagulase-negative Staphylococci and Streptococci species in blood infections among patients of hematological malignancies [15].

Other known microbial opportunistic pathogenic groups such as *Staphylococcus aureus* (9.75%), *Pseudomonas aeruginosa* (7.31%), *Acinetobacter baumannii* (4.87%), *Enterobacter cloacae* (4.87%), and *Proteus mirabilis* (4.87%) were detected at varying rates among the BSI samples of patients. *Pseudomonas aeruginosa* and *Acinetobacter baumannii* are known for drug-resistant pathogens causing serious blood infections in individuals with low immunity. Leal et al. [16] reported that these organisms were isolated from the blood cultures in case of severe bacteremia and septicemia involved in leukemia patients. Several other researchers have also found these organisms in molecular-based methods, but not in culture-based techniques [17]. Hence, this reinforces the need for molecular-based techniques like NGS in identifying pathogens in BSIs which are not covered in routine blood cultures.

Interestingly, four of the control samples with no signs of bloodstream infections showed positive reaction in sequence for coagulase-negative Staphylococci, *Staphylococcus aureus*, and *S. epidermidis*. However, it is uncommon to have bloodstream infections in healthy blood donors, and the population structure of the microorganism indicates a possible skin microflora contamination during venipuncture, as these organisms are common microflora of the skin. Hall and Lyman [17] had earlier reported the contamination of skin microflora in case of blood culture and other molecular techniques. Hence, it is unlikely that these microorganisms had resulted in bloodstream infections in such blood donors. Further, history records of the blood donors state no signs and symptoms of any infections in the previous fifteen days and hence could be attributed to venipuncture artefacts.

Of the total number of reads, among bloodstream infections in patients, 51.21% of the cases had Gram-negative infection, while control samples had no such findings. About 34.14% of the cases among patients showed Gram-positive infection, while 14.63% of the cases showed polymicrobial bloodstream infections. In the case of control samples, only Gram-positive infection was found in all cases (100%) (Figure 2). Most (90%) of the identified reads represented opportunistic human pathogens in HM patients, while there was normal human microbiota in control samples. Trecarichi et al. [12] had earlier reported that BSI are mainly caused by the Gram-negative group of microorganisms and more so in cancer patients. Their study showed the most frequently isolated Gram-negative bacteria were *E. coli* and *K. pneumoniae*, which is in line with our studies, wherein *E. coli* was 19.51% and *K. pneumoniae* was 17.07% and were the most frequently isolated Gram-negative bacteria. Generally, the Gram-negative members are more virulent in causing infections in humans; hence, they are more frequently isolated. However, infection with both Gram-negative and Gram-positive organisms (polymicrobial) is also noted in the case of Trecarichi et al. [12]. Similar to these results, the present study also documents polymicrobial infections in bloodstream infections of the patients studied, making it more complex for treatment and management. However, control samples showed no polymicrobial infection, instead only Gram-positives—indicating possible contamination-associated microorganisms.

Chronic lymphoid leukemia showed the highest percentage of both Gram-negative infections (42.86%) and Gram-positive infections (35.71%) than all other types of leukemias. These patients were also detected with polymicrobial infection in 50% of cases, indicating the complexity of the bloodstream infections in leukemia patients and difficulty in its antimicrobial management. Andersen et al. [18] and coworkers showed that CLL patients are more prone to bloodstream infections owing to the severity of the disease and therapeutic options available. Several other workers also reported a high rate of bloodstream infections in CLL patients [18, 19]. Chronic myeloid leukemia had the second-highest rate of bloodstream infection with 33.33% Gram-negative and 28.57% of Gram-positive infection with 33.33% case rate of polymicrobial infections. Control samples (blood donors with no hematological malignancies) showed only Gram-positive infections with no signs of Gram-negative or polymicrobial infections (Figure 2). This indicates the possibility of contamination of the blood samples during venipuncture and the results not attributed to blood infections.

In recent years, there has been an increase in the incidence rates of hematological malignancies, particularly among the Central Province, Eastern region, and Northern region populations [20]. Hematological malignancies, by virtue of their treatment modalities, put the patients at higher risk of microbial infections. A broad range of infectious agents—bacteria, fungi, protozoa, and viruses—can infect locally or systemically. Unscrupulous use of a wide array of antibiotics for prophylaxis in cancer patients has led to the development of drug-resistance among many bacterial species. Controlling immune deficit, preventing microbial infections, and implementing antibiotic stewardship are some of the key aspects of the management of hematological malignant patients. For favorable outcomes, accurate diagnosis of the infection and timely initiation of appropriate antibiotic therapy are imperative. Hence, NGS-based detection methods can pave the way for the detection of BSIs in such patients timely and specifically.

## 8. Conclusions

The present study detects that there exists a high rate (82%) of bloodstream infections among patients of hematological malignancies in Saudi Arabia. Gram-negative opportunistic, Proteobacteria group members such as *Escherichia coli*, *Klebsiella pneumoniae*, and *Pseudomonas aeruginosa* are highly detected in these cases, while normal blood donors with no hematological malignancies showed insignificant microbial population. Most of the bloodstream infections in patients were characterized by polymicrobial infections unlike control in samples, indicating the complexity of the infection and subsequent antimicrobial therapy. Next-generation sequencing showed robust, fast, and specific identification of infectious agents in bloodstream infections in hematological malignancies, indicating the possibility of its application in routine follow-up of such patients for infections. However, the limitations of this study, such as low sample size and restricted geographic location of the sampling, may be taken into account when further research work is designed to include more samples with representation from all the geographic regions. Future research is required on the study of molecular characterization of multidrug resistance among bloodstream infections causing microorganisms identified in such patients using next-generation sequencing.

## Figures and Tables

**Table 1:** Characteristics of patients with hematological malignancy in this study.

Patient characteristic	Value (range, %age)
Median age (years)	41 (18-74)
No. (%) of patients with diagnosis	
Acute myeloid leukemia	9 (21.95%)
Chronic myeloid leukemia	10 (24.39%)
Acute lymphatic leukemia	15 (36.58%)
Chronic lymphoid leukemia	5 (12.19%)
Non-Hodgkin lymphoma	1 (2.43%)
Hodgkin lymphoma	1 (2.43%)
WBC (/*μ*l)	497 (8-3800)
Neutrophil (/*μ*l)	109 (5-850)
Fever period (days)	4.3 (1-13)

**Table 2:** Bacterial species detected in blood samples from hematological malignancies.

Hematologic malignancies (*n* = 50)	No. (%)	Control (*n* = 50)	No. (%)	*p value*
Blood stream infection	41 (82)	Blood stream infection	04 (8.0)	<0.05

Gram-negative group	24 (58.53)	Gram-negative group		
*Escherichia coli*	08 (19.51)	—	—	<0.05
*Klebsiella pneumoniae*	07 (17.07)	—	—	<0.05
*Pseudomonas aeruginosa*	03 (7.31)	—	—	<0.05
*Enterobacter cloacae*	02 (4.87)	—	—	NS
*Acinetobacter baumannii*	02(4.87)	—	—	NS
*Proteus mirabilis*	02 (4.87)	—	—	NS

Gram-positive group	17 (43.5)	Gram-positive group		
*Coagulase -ve staphylococci*	07 (17.07)	*Coagulase -ve staphylococci*	01 (2.0)	NS
*Staphylococcus aureus*	04 (9.75)	*Staphylococcus aureus*	01 (2.0)	NS
*Viridians streptococci*	06 (14.63)	*Staphylococcus epidermidis*	02 (4.0)	NS

NS: nonsignificant; *p* < 0.05 is statistically significant.

## Data Availability

The data used to support the findings of this study are included within the article.
